# Do we know how scabies outbreaks in residential and nursing care homes for the elderly should be managed? A systematic review of interventions using a novel approach to assess evidence quality

**DOI:** 10.1017/S0950268819001249

**Published:** 2019-08-05

**Authors:** E. J. Morrison, J. Middleton, S. Lanza, J. E. Cowen, K. Hewitt, S. L. Walker, M. Nicholls, J. Rajan-Iyer, J. Fletcher, J. A. Cassell

**Affiliations:** 1 Brighton and Sussex Medical School, Brighton, UK; 2 School of Life Sciences, University of Sussex, Brighton, UK; 3 Surrey and Sussex HPT (South East), Public Health England, County Hall North, Chart Way, Horsham, West Sussex RH12 1XA, UK; 4 Faculty of Infectious and Tropical Diseases, London School of Hygiene and Tropical Medicine, London, UK

**Keywords:** Elderly medicine, infectious disease control, outbreaks, residential care, scabies

## Abstract

Currently no national guidelines exist for the management of scabies outbreaks in residential or nursing care homes for the elderly in the United Kingdom. In this setting, diagnosis and treatment of scabies outbreaks is often delayed and optimal drug treatment, environmental control measures and even outcome measures are unclear. We undertook a systematic review to establish the efficacy of outbreak management interventions and determine evidence-based recommendations. Four electronic databases were searched for relevant studies, which were assessed using a quality assessment tool drawing on STROBE guidelines to describe the quality of observational data. Nineteen outbreak reports were identified, describing both drug treatment and environmental management measures. The quality of data was poor; none reported all outcome measures and only four described symptom relief measures. We were unable to make definitive evidence-based recommendations. We draw on the results to propose a framework for data collection in future observational studies of scabies outbreaks. While high-quality randomised controlled trials are needed to determine optimal drug treatment, evidence on environmental measures will need augmentation through other literature studies. The quality assessment tool designed is a useful resource for reporting of outcome measures including patient-reported measures in future outbreaks.

## Introduction

Scabies is a common and disabling dermatological condition caused by infestation with the mite *Sarcoptes scabiei* [1–3]. Designated as a Neglected Tropical Disease by the World Health Organization, it remains an important public health issue in the United Kingdom (UK) [4], especially in institutional settings such as residential or nursing care homes for the elderly (RNC), where outbreaks are typically prolonged, recognition is often delayed and management is highly challenging [1, 5, 6].

Scabies is mainly spread through direct skin to skin contact, and less commonly through fomites. Symptoms tend to present 4–6 weeks after exposure due to a delayed hypersensitivity reaction to the scabies mites' faeces and eggs, or within a week if after repeated infestation [3, 7, 8]. The classical presentation is an erythematous papular rash and pruritus, which is typically worse at night [3, 9].

The elderly, young and immunocompromised are particularly vulnerable to scabies [10, 11], and RNC are especially susceptible to institutional outbreaks [12]. The typical distribution of clinical signs may differ in the elderly [8, 13, 14]; residents may be asymptomatic or have subtle signs, adding to difficulty in diagnosis in this population and contributing to delayed diagnosis. Residents with dementia are at increased risk of scabies [14]. The highly contagious crusted form of scabies is also difficult to recognise in RNC residents, where it may occur more commonly due to misdiagnosis and treatment with corticosteroids. It presents with a hyperkeratotic rash which may lack the characteristic itch and be more easily transmitted due to high mite burden [9, 15].

### Management of scabies outbreaks

An outbreak of scabies can be defined as two or more cases of classical scabies, or a single case of crusted scabies, linked by time in the same environment [16]. The control of outbreaks in RNC is time-consuming and cost-intensive, requiring mass treatment of infected cases and contacts. It is complicated by issues including atypical presentation, close proximity of residents, carers and visitors, mental capacity issues, financial responsibility for treatment and logistical barriers of mass treatment [15, 17, 18]. Here we focus on outbreaks in RNCs only.

In the UK, existing national guidelines focus on management of individual cases, and none exist for scabies outbreaks in RNC [19]. In England, local Health Protection Teams (HPTs) predominantly provide advice on the control of institutional outbreaks using locally developed guidelines which are highly variable in their recommendations [17]. No systematic review has been undertaken of the effectiveness of interventions in outbreaks.

### Current drug treatments for scabies outbreak management

Drug treatments for scabies include topical acaricides (such as benzyl benzoate, crotamiton, lindane, malathion, sulphur and permethrin) and oral ivermectin, an anti-parasitic. A Cochrane review of scabies treatments recommended topical permethrin, but concluded ivermectin was an effective oral treatment [20]. Current National Institute for Health and Care Excellence (NICE) guidelines for the treatment of individual cases of scabies recommend permethrin 5% cream as first line treatment, and malathion 0.5% aqueous liquid if permethrin is contraindicated [19]. Treatment is applied to the whole body of all household members and their close contacts, even if asymptomatic, left on for 8–24 h and a second application 1 week later is recommended [19]. Oral ivermectin is unlicensed but can be used on a named-person basis [21]. Itch frequently persists following therapy, and may not indicate treatment failure; symptomatic treatment is recommended when needed [19].

### Environmental management measures

There is some evidence of indirect scabies transmission through fomites (clothing, bedding, furniture, carpet) [22], suggesting environmental management measures including cleaning of RNCs may have a role in preventing transmission. This transmission is thought to be rare with classical scabies but may occur with the crusted form [1], though limited due to mites' inability to survive off human skin for long periods [2, 9].

NICE guidelines recommend machine washing clothes, towels and bed linen at 50 °C on the day of first treatment and referral of institutional outbreaks to the HPT to advise on infection control measures [19].

### How can outbreak outcomes be measured? The need for a framework

Outbreak measures must address the key dimensions described above: timely diagnosis, drug treatment, environmental management and symptomatic care.

Given the diagnostic and management challenges in RNC described above, these must describe all points in the recognition and treatment pathway in order to assess efficacy of interventions, patient experience and how best to measure outbreak duration and delayed recognition or intervention.

**Fig. 1.**
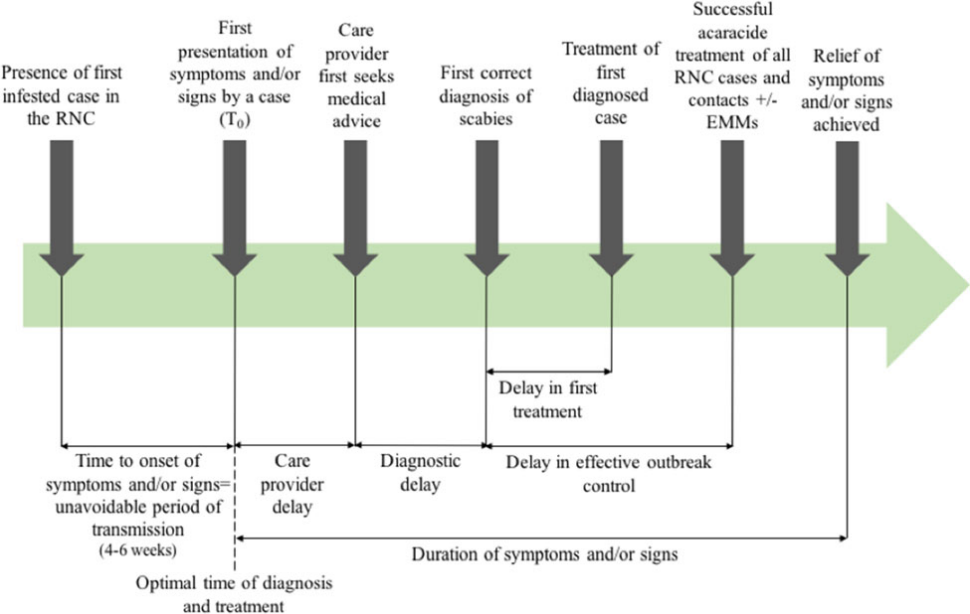
Outbreak measures in the timeline of a scabies outbreak in a RNC, including points in the diagnosis and management timeline where delay may occur.

Outcome measures used in previous systematic reviews have included the rate of treatment failure (persistence of original scabies lesions, development of new lesions or identification of mites on a skin scraping), number of new cases following treatment [20], or need for repeat implementation of environmental infection control measures. Figure 1 shows the points at which delay may occur, which will inform this review.

In order to provide high-quality care, patients' perception of their care must be considered. Patient-Reported Outcome Measures (PROMs) assess the efficacy of a treatment from the patients' perspective. PROMs in scabies infestation and management may include duration or severity of itch, quality of sleep and rate of secondary infection. Patient-Reported Experience Measures (PREMs) evaluate the patients' views of their experience while being treated [23].

We undertook a systematic review of evidence on the effectiveness of interventions for the management of scabies outbreaks in RNC.

## Aims

To systematically review evidence and determine how to best manage scabies outbreaks in RNC. We address two empirical research questions:

1. What is the most effective drug treatment for scabies outbreaks in residential or nursing care homes for the elderly?
2. Which environmental infection control measures should be undertaken to prevent further transmission and prolonged infestation following an outbreak in this setting?

To inform future research, we also address methodological research questions:

1. What are the most useful outcome measures in assessing the effectiveness of scabies management measures?
2. How should delayed diagnosis and treatment be measured?

For the purposes of the current review, ‘elderly’ is defined as a mean age of residents of over 65 years, where described.

## Materials and methods

### Search strategy

A literature search of four databases (PubMed, Cinahl, Embase and Web of Science) was performed on 7 January 2017 and repeated on 19 July 2017, using the terms ‘(((scabies OR crusted scabies OR sarcoptes scabiei OR scabies mites)) AND (residential home OR care home OR residential facility OR long term care facility OR nursing home)) AND (treatment OR benzyl benzoate OR permethrin OR ivermectin OR malathion OR lindane OR sulfur OR scabicide lotion OR infection control OR washing OR vacuum OR hoover OR cleaning OR carpet OR upholstery OR bedding OR clothes OR isolation OR gloves OR aprons OR care home closure)’.

Citations were retrieved from inception to the date of search. Additional papers were identified using Google Scholar citation searching and PubMed related articles. The PRISMA statement was followed [24]. The search was carried out by two separate reviewers (EJM and JEC). Search results were imported and stored in EndNote Web, duplicates were removed and articles that were not relevant, judged by titles and abstracts, were excluded. All study designs were eligible for inclusion.

### Inclusion and exclusion criteria

Articles were eligible for inclusion if they met two criteria: (i) studies of scabies outbreak management in RNC, and (ii) studies describing either drug treatment or environmental infection control measures implemented. Non-English language, animal studies and those describing a scabies outbreak in other settings, were excluded. Both reviewers screened search results for compliance with the inclusion and exclusion criteria; disagreements were discussed and resolved by consensus.

### Quality assessment

All studies to be included in the review were assessed for quality by the first author. We intended to assess the quality of evidence and overall strength of recommendations using the GRADE (Grading of Recommendations, Assessment, Development and Evaluations Working Group) criteria [25]. However, GRADE differentiates poorly between the quality of non-comparative observational studies, classifying them all as low or very low quality evidence, so that any recommendations for the management of scabies outbreaks in RNC would be weak based on the reports identified (see the ‘Results’ section).

A quality assessment tool was therefore developed from the STROBE (Strengthening the Reporting of Observational Studies in Epidemiology) checklist [26], to capture the quality of observational data for this review. A suite of 17 outcome measures considered suitable for these outbreaks was developed based on this in the absence of a standard description.

### Data extraction

Data items extracted included details of the population and setting, number of cases identified, drug treatments and environmental infection control measures implemented and outcome measures reported.

Primary outcome measures were treatment failure of a confirmed case or need for repeat environmental measure implementation. The secondary outcome measure was duration of outbreak (defined as time from identification of infestation of the index case to successful treatment resulting in no new or repeat cases). Adverse events were considered serious if life threating or resulted in death or hospitalisation. Side effects including those which required discontinuation of treatment or caused patient discomfort or dissatisfaction were recorded.

The timescale of the outbreaks including date of first presentation of symptoms or signs by a case, first correct diagnosis of scabies, treatment of first diagnosed case and successful treatment of all RNC cases were extracted where available in order to describe duration of any delays in outbreak management.

## Results

### Literature search

**Fig. 2.**
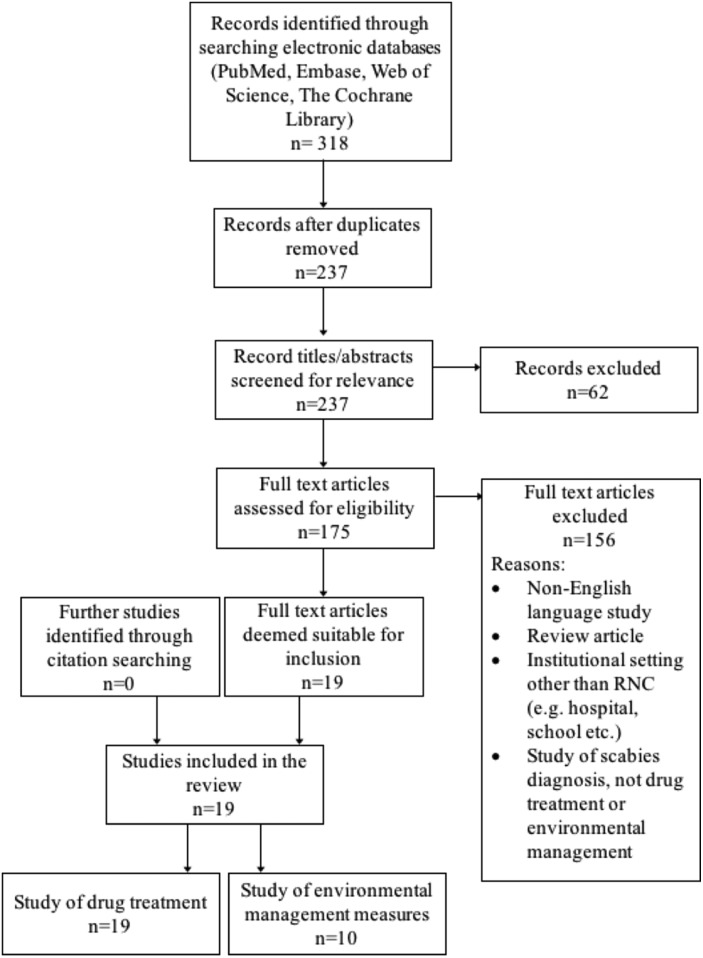
Flow of study selection at each stage from identification to final inclusion, including study numbers for both drug treatment and environmental management measures.

A summary of the literature search is shown in Figure 2. Nineteen studies were included in the review, dating back to 1983. All evaluated the drug treatment of scabies in RNC, and 10 also assessed one or more environmental management measure. The included studies are detailed in Table 1. Nearly all were excluded due to not presenting primary data on an outbreak. These ranged from discussion pieces, editorials and educational articles. A few reported outbreaks in settings that did not meet our inclusion criteria.

**Table 1.** Summary of all studies included in the review, including outcome measures described in reports

Author	Year	Country	Setting (number of scabies cases diagnosed)	Outcome measures	Drug treatment/environmental measures/both
Andersen *et al*. [27]	2000	Norway	Three nursing homes (24)	Number of cases before and after treatment, repeat cases	Both
Apap *et al*. [28]	2012	Malta	Residential home – treated in hospital (unknown)	Time from diagnosis to successful treatment of index case	Drug
Barkwell *et al*. [29]	1997	Canada	Long-term care facility (unknown)	Outbreak duration	Drug
Burns *et al*. [30]	1987	UK	Residential home (4)	Number of new cases after treatment	Both
Chan *et al*. [31]	2000	Hong Kong	Old-age home (9)	Time from symptoms to successful treatment of index case	Drug
Dannaoui *et al*. [32]	1999	France	Nursing home (7)	Treatment failure and repeat cases, outbreak duration	Both
De Beer *et al*. [16]	2006	South Africa	Long-term care facility (57)	Average duration of rash before treatment initiation, outbreak duration	Both
Hetland *et al*. [33]	1987	USA	Nursing home intermediate care facility (41)	Time from first symptoms to diagnosis, treatment failure rate	Both
Ladbury *et al*. [34]	2012	Netherlands	Four elderly care homes as part of a wider community outbreak (26)	Number of repeat cases or new cases after treatment	Drug
Millership *et al*. [35]	2002	UK	Homes for the elderly mentally ill (19)	Number of repeat cases or new cases after treatment	Drug
Moberg *et al*. [36]	1984	Sweden	Nursing home (14)	Treatment failure and repeat cases	Drug
Paasch *et al*. [37]	2000	Germany	Three residences for the elderly (252)	Treatment failure and repeat cases	Both
Papini *et al*. [38]	1999	Italy	Two nursing homes (91)	Outbreak duration	Drug
Parish *et al*. [39]	1983	USA	Three nursing homes (unknown)	Need for repeat treatment	Drug
Paules *et al*. [40]	1993	USA	Nursing home (6)	Treatment failure and repeat cases, repeat management measures, outbreak duration	Both
Sullivan *et al*. [41]	1997	Australia	Nursing home (33)	Treatment failure and repeat cases	Both
Van den Hoek *et al*. [42]	2008	Netherlands	Nursing home (5)	Cases remaining symptom free at 3 months	Both
Wilson *et al*. [43]	2001	USA	Long-term care geriatric facility (15)	Time to diagnosis, treatment failure	Drug
Yonkosky *et al*. [44]	1990	USA	Three nursing homes (202)	Treatment failure and repeat cases	Both

### Quality assessment

Table 2 shows results of our assessment of included studies using the STROBE derived quality assessment tool. Delays in management and outbreak duration were identified through reporting of dates of outbreak identification, management measure implementation and outbreak conclusion. No case report described all 17 points, and seven reports were particularly poor [28, 29, 31, 33, 38, 39, 43], detailing less than 10 points.

**Table 2.** Results of quality assessment tool adapted from the STROBE checklist for observational studies

Outcome measure	Author																		
	[27]	[28]	[29]	[30]	[31]	[32]	[16]	[33]	[34]	[35]	[36]	[37]	[38]	[39]	[40]	[41]	[42]	[43]	[44]
Study design and outbreak identification:																			
1. Description of study design	−	+	−	+	+	−	+	−	+	−	+	−	−	+	+	−	+	−	−
2. Description of setting	+	+	+	+	+	+	+	+	+	+	+	+	+	+	+	+	+	+	+
3. Description of how outbreak identified	+	+	−	+	+	+	+	+	+	+	+	+	+	+	+	+	+	−	−
4. Method of case diagnosis described	+	+	−	+	+	+	+	+	−	+	+	+	+	+	+	+	+	+	+
5. Number of individuals assessed for scabies	+	−	−	+	−	+	−	+	−	−	+	+	+	+	+	+	−	−	+
6. Characteristics of cases (demographic, clinical, social)	−	−	+	+	−	−	+	−	+	+	+	+	+	−	+	−	+	+	+
7. Number of cases reported	+	−	−	+	+	+	+	+	+	+	+	+	+	−	+	+	+	+	+
Efficacy of management measure:																			
8. Full description of treatment including who treated and why	+	−	+	+	−	+	+	+	+	+	+	+	+	+	+	+	+	+	+
9. Full description of EMMs implemented	+	−	−	+	−	+	+	+	−	−	−	+	−	−	+	+	+	−	+
10. Number of repeat cases observed after treatment	+	−	+	+	−	+	+	+	+	+	+	+	−	−	+	+	+	+	+
11. Number of new cases observed after treatment	+	−	−	+	−	+	+	+	+	+	+	+	−	−	+	+	+	+	+
12. Adverse events or side effects reported	−	−	+	−	−	+	−	−	−	+	+	+	−	+	−	+	−	−	+
Management delay:																			
13. Date of outbreak identification reported	+	−	+	−	−	+	+	−	+	+	−	−	+	−	+	−	+	+	+
14. Date of mass treatment±environmental management implementation	−	−	+	−	−	+	+	+	−	+	−	−	−	−	+	−	+	−	−
15. Date outbreak declared over reported	+	−	+	−	−	+	+	−	+	+	−	−	+	−	+	−	+	+	−
Patient Experience measures:																			
16. PROMs/PREMs reported (severity/duration of itch, quality of sleep, secondary infection etc.)	−	−	−	−	−	−	−	−	−	−	−	−	−	−	−	−	−	−	−
17. Description of symptomatic treatment given	−	−	+	−	+	−	−	−	−	−	−	+	−	+	−	−	−	−	−
Total	11	4	9	11	6	13	13	10	10	12	11	12	9	8	14	10	13	9	11

+ Represents criteria achieved. − Represents criteria not achieved.

### Outcome measures

The reporting of outcome measures varied markedly. Two case reports [28, 31] only discussed treatment of a single index case after hospital admission for treatment and did not further describe the outbreak. Five reports did not describe the rate of treatment failure [28, 29, 31, 38, 39], and three did not report the number of new cases diagnosed after treatment [28, 29, 31]. No definitions of treatment failure were given. Seven articles did not report outbreak duration [28, 30, 31, 33, 36, 37, 44]. Notably, no PREMs or PROMs were reported in any outbreak.

### Outbreak management delays

Delay in diagnosis or in successful treatment was described in all but one case report in which the time scale of the outbreak was not specified [38]. Twelve described a delay in diagnosis [16, 27, 28, 30, 31, 33, 34, 36, 37, 40, 42, 43], either stating there was a delay or reporting the duration of time cases had symptoms before a diagnosis of scabies was made. Fifteen reported a delay in effective treatment [16, 27, 29, 30, 32, 33, 35–37, 39–44], due to treatment failures or new cases following treatment. In eight studies [16, 27, 28, 31, 33, 36, 39, 40], misdiagnosis was reported. Though not all articles specified the duration of delay; where reported, the median duration from onset of symptoms to diagnosis was three months, and median delay in effective treatment was 9 months.

### Drug treatments

Over 800 cases of scabies were described in 19 studies. Drug treatment of cases and prophylaxis of contacts (asymptomatic residents, staff and family) varied widely, as did success. Common drug regimens were either topical treatment alone, topical treatment in combination with oral ivermectin, or ivermectin alone. A summary is provided in Table 3.

**Table 3.** Summary of drug treatments implemented in reports. Alternative treatments used due to new cases arising following treatment or unresolved cases. Note some RNCs reported the use of different drug treatments for different residences affected

	Number of reports in which treatment used first line	Number of reports in which alternative needed	Alternative treatment used
Topical treatments			
Lindane	7	4	Ivermectin, crotamiton, permethrin
Permethrin 5% cream	4	1	Ivermectin
Benzyl benzoate	3	1	Permethrin
Crotamiton	3	2	Ivermectin, permethrin
Tenutex	1	0	
Allethrin spray	1	0	Ivermectin for crusted scabies
Topical treatment in combination with oral treatment			
Lindane + ivermectin	1	1	Ivermectin + permethrin
Malathion, permethrin + ivermectin	1	0	
Permethrin + ivermectin	1	0	
Oral treatment only			
Ivermectin	3^a^	0	

Tenutex = DDT 0.5%, disulphiram 2%, benzyl benzoate 22.5%; commercial purified form of GBHC (gamma benzene hexachloride) is lindane, therefore results combined.

a Staff and contacts were treated with topical permethrin in all three reports.

Permethrin cream was used first in four reports [16, 37, 38, 43], which resulted in two repeat cases in one report and three repeat cases in another. These resolved with further permethrin application or oral ivermectin therapy. In two studies other topical treatments were used initially (Lindane, benzyl benzoate and crotamiton) with high levels of treatment failure, before permethrin was used, resulting in resolution of the outbreak [40, 44]. Of the 14 case reports where topical treatments were used alone, oral ivermectin was required in three for outbreak resolution [29, 32, 43]. In two case reports [37, 38], crusted scabies cases were identified and treated differently from typical scabies, commonly with a combination of topical treatment and oral ivermectin, which successfully ended the outbreak. Notably, in all case reports in which oral ivermectin was used, it resulted in resolution of the outbreak [28, 29, 32, 34, 35, 41–43].

In 13 case reports [16, 28, 30, 32–37, 39, 40, 42, 44], all residents, staff and visiting contacts were treated. The rest varied between residents only [29, 38, 41], confirmed cases [43] or confirmed cases and close contacts [27]. One report did not detail who was treated [31]. Two reports also described a different treatment regimen for residents (ivermectin) and staff or other contacts (permethrin cream) [34, 35].

Excess death was reported by Barkwell *et al*. [29], in 6 months following ivermectin treatment. Four other reports of ivermectin use did not report this [32, 35, 37, 41]. There were six reports of increased pruritus following scabies treatment with ivermectin [35, 41], Tenutex [36], allethrin spray [37], lindane [39] and permethrin [44].

### Symptomatic relief

One report described the use of crotamiton cream for symptomatic relief of persistent pruritus following treatment [39], and another described re-treatment if pruritus persisted beyond initial anti-scabies treatment [37]. In two outbreaks crotamiton was used as part of the treatment regimen [29, 31]; while other reports did not describe any symptomatic treatment for itch.

### Environmental management measures

Ten reports described implementation of environmental measures to aid in outbreak control. Description of these varied widely, with some reports outlining protocols and others briefly stating RNC cleaning took place. The most commonly reported measures were ‘spray or disinfection of the infested persons’ environment’ and ‘washing of clothes’.

A summary of environmental management measures reported is presented in Table 4. Only two reports described protocols for the timing of these in relation to treatment [37, 41]. Two others described repeat cleaning or infection control measures following treatment [27, 33]. Despite reporting requiring additional drug treatment for treatment failures in seven of the 10 articles, only one reported repetition of environmental management measures in association with repeat treatment [32].

**Table 4.** Summary of environmental management measures implemented in reports

Environmental management measure	Author									
	[27]	[30]	[32]	[16]	[33]	[37]	[40]	[41]	[42]	[44]
Isolation of cases	+	+	+	+	−	−	−	−	−	−
Use of gowns±gloves	+	+	−	+	+	+	−	−	−	−
Handwashing between care	+	−	−	−	−	−	−	−	−	−
Long sleeves worn	+	−	−	+	−	−	−	−	−	−
Washing of clothes	+	−	+	+	+	+	+	+	+	+
Spray or disinfection of environment	+	+	−	+	+	+	+	+	+	+
Upholstery/bed linen washed, frozen or bagged	+	−	+		+	+	+	+	−	+
Visitor ban	−	−	−	+	−	−	−	−	−	−
Suspend admissions	−	−	−	+	+	−	−	−	−	−

+ Represents measure implemented. − Represents measure not implemented or not described.

Where information regarding a measure was not provided in the text, the report was scored as not achieving said point (−).

## Discussion

This systematic review reveals a moderate number of reports of the management of scabies outbreaks in RNC, but the absence of comparative studies assessing either drug treatment or environmental management. Permethrin was reported to be an effective treatment for scabies outbreaks in RNC, resulting in just five repeat cases. While lindane was also a commonly used topical treatment, further anti-scabies treatment was required in four of the seven outbreaks in which it was used. It was not possible to determine the effects of environmental management measures on any of the outcome measures in this review due to the lack of comparative studies. The quality of the data presented is low, and outcome measures used in previous literature (e.g. rate of treatment failure or duration of outbreak) were often missing. This limits the value of this observational evidence, which is much weaker than it need be. Outcomes within studies are poorly documented, with PROMs and PREMs almost entirely absent.

Consequently, no clear recommendations can be made about the effectiveness of drug or environmental interventions for outbreaks in RNC (beyond NICE guidelines for individuals). The recommendation in recent German guidelines [15], for permethrin with ivermectin as an alternative appear reasonable pending better evidence, especially given that individual comparison of lindane with permethrin shows it to be inferior [45]. There is clearly an urgent need for comparative, preferably randomised studies of the various drug treatments used, and these should include detailed description of all elements in the pathway from recognition of an index case through to evaluation of the effectiveness of outbreak control.

Despite the poor quality of data reporting, it was notable that delay in recognition or treatment of outbreaks was almost universally reported, with almost half of reports describing initial misdiagnosis. This is consistent with recent reports of RNC experience [6]. While RNC is a difficult setting to access for outbreak research [46], which is necessarily done in a hurry, high-quality studies of interventions for infectious diseases in care homes can be achieved [14, 47].

The poor data quality we observed also highlights the importance of using comprehensive and consistent data collection strategies to describe and analyse delay. This could be through the framework we present in Figure 1, which has been operationalised in our multi-outbreak report describing the clinical features of outbreaks in RNC [14]. The quality assessment tool we adapted from the STROBE checklist used to capture the quality of observational data in this review may also be more widely applicable, to better differentiate studies where randomised controlled trials or controls are lacking.

It is less clear how an evidence base for environmental measures should be developed, especially given the uncertainty about drug treatments with which they will necessarily interact. Experiments on the survival of the scabies mite off its human host under the range of environmental conditions found in the RNC setting could inform the maximum time period after contact when decontamination is worth considering, while veterinary data may address in part the likely increased transmissibility of crusted scabies. A recent review by Arlian *et al*. [48] concluded that disinfection should not be necessary and isolation of the bed, bedding and clothing for 48 h should be sufficient. However, this would be challenging in RNC, and little evidence exists to support this from human studies. This is an urgent question for RNC – one report identified drug treatment as just one-fifth of the cost associated with the outbreak, with the majority of cost due to staff overtime, disposable gowns and gloves, cleaning supplies and laundry services [16].

As so often in reports of infectious disease outbreaks, these case reports did not systematically address PREMs which are essential in the development of guidelines as they assess patients' objective experience of care and determine acceptability of a management strategy from a patient perspective. Although PREMs may be difficult to assess in this setting where many residents may be cognitively impaired, they must still be considered and reported where possible. In six reports increased pruritus was noted as a side effect following scabies treatment with a range of drugs. It is recognised that itch may persist for several weeks following scabies treatment [11], yet few reports recognised this and failed to describe whether symptomatic treatment was given alongside anti-scabies treatment. Drug treatments used for itch include topical crotamiton, topical hydrocortisone or oral antihistamines [19]. In order to address resident comfort and reduce suffering, symptomatic relief should be considered in future evidence-based guidelines.

In many outbreaks all residents, staff, family and contacts were treated and multiple treatment failures were ascribed to the lack of a highly-coordinated management plan. Whilst there are no UK national guidelines on scabies outbreaks in RNC [17], NICE guidelines for other cases of scabies infestation recommend simultaneously treating all household and close contacts [19]. Despite this advice, a recent systematic review by Fitzgerald *et al*. [49] found there is currently no evidence for the use of prophylactic treatment to prevent infestation in contacts. Patterns of exposure are likely to differ between family households and RNC, and the question of contact treatment should be addressed in future comparative trials of drug treatments.

One report by Barkwell *et al*. [29] described an increase in mortality in 6 months following ivermectin therapy in RNC residents. This cross-sectional study comparing deaths in a fixed period after ward level topical treatment and ivermectin mass treatment in two long stay wards has been heavily criticised due to the failure to control for factors leading to ward allocation and other confounders including other treatments, and it is not possible to conclude any increase in mortality is the result of ivermectin therapy [50, 51]. These results have not been replicated in any other study of ivermectin in RNC [32, 35, 37, 41, 51], a drug which is used worldwide in the mass treatment of river blindness (onchocerciasis) and serious adverse effects reported are rare [21].

This study reports only peer reviewed studies and it is possible that outbreak reports in the grey literature of similar quality may have been missed by our search strategy. We only included studies reported in English, and it is possible that comparative studies were missed. We did not review veterinary studies or experiments on mite survival which may be needed to specify and describe environmental interventions.

Overall, this review has highlighted a lack of comparative studies assessing the optimal drug treatment or necessary environmental management measures for outbreak control in RNC. Current observational evidence is weak and the evidential value of future studies could be improved through better reporting of outbreak outcome measures, such as time to diagnosis and time to effective treatment, as these incorporate any diagnostic delay or delay in effective treatment, which was common within reports. Patient-reported outcome and experience measures of treatment are important in the consideration of future national guidelines for managing these outbreaks and should be prioritised in future studies.
